# Novel Strategies for Meat Processing, Preservation, and Quality Improvement

**DOI:** 10.3390/foods15071135

**Published:** 2026-03-25

**Authors:** Ivan A. Garcia-Galicia, Mariana Huerta-Jimenez

**Affiliations:** 1 Centro de Enseñanza, Investigación y Extensión en Ganadería Tropical, Facultad de Medicina Veterinaria y Zootecnia, Universidad Nacional Autónoma de México, Carr. Fed. Martínez de la Torre-Tlapacoyan km 5.5, Tlapacoyan 93650, Veracruz, Mexico; 2 Facultad de Zootecnia y Ecología, Universidad Autónoma de Chihuahua, Periférico Francisco R. Almada km 1, Chihuahua 31453, Chihuahua, Mexico; mhuertaj@uach.mx

## 1. Introduction

Meat is a cornerstone in human nutrition worldwide, providing essential high-quality proteins, vitamins, minerals, and healthy fats. Nevertheless, many traditional meat products often fail to fulfil technological, sensory, and nutritional demands, mainly due to inadequate processing and preservation.

These challenges highlight the need for research on interventions that considerably improve meat’s properties. The search for innovative solutions to increase meat quality as well as offer health benefits and ensure sustainable production is now compulsory due to the convergence of global challenges involving food safety, environmental sustainability, and drastic changes in consumer preferences. These innovations should not be just temporary solutions but consistent responses to ensure the viability of the meat industry in an increasingly demanding and regulated market [1,2].

The meat industry must provide consistent sensorial meat characteristics such as flavor, texture, and color. At the same time, the demand is growing for “clean-label” products that do not contain chemical preservatives or enhancers. This demand necessitates the development of natural bio-preservatives using plants or their components and active packaging systems that naturally extend shelf life [3,4]. A current trend is incorporating cost-effective, safe, and efficient natural ingredients and processes into meat production, which are essential for improving meat beyond its basic nutritional value. These interventions can substantially improve the yield, texture, and overall quality of meat and meat products, while meeting consumer needs [3].

Alternatively, technologies such as high-intensity ultrasound could be implemented in production lines to increase the tenderization and juiciness of meat, overcoming the limitations of traditional processing methods that often damage meat’s cell structure. Furthermore, global outbreaks of pathogenic microorganisms continue to occur. Conventional techniques sometimes fail against antibiotic-resistant strains; as such, alternative methods, for example, combining ultrasound with traditional methods, are essential for inactivating microbes without compromising the nutritional, sensorial, or technological value of meat [5,6].

Traditional meat production has several environmental impacts, among which meat packaging is a global concern due to the use of non-recyclable and fossil-fuel-based plastics. New strategies aim to reduce microplastic pollution and their impact on the planet and human health. These strategies are centered on the use of natural packaging that reduces environmental issues and meets the requirements for high-performance packaging that prevents food safety risks, spoilage, and massive food waste [7,8].

## 2. Overview of Published Articles

The articles published in this Special Issue provide influential innovations, focusing on meat processing techniques and adding natural ingredients to improve the quality and technological attributes of meat. One article offers a transformative approach to meat production centered mainly on cultured meat technology as a sustainable, healthy method that avoids animal suffering.

Nurul Alam and collaborators (Contribution 1) fabricated hybrid electrospun nanofiber active packaging (HENF) using green tea extract. HENF lengthens beef’s shelf life and increases meat quality, addressing environmental concerns related to packaging waste. The physicochemical and sensory qualities of Hanwoo beef in HENF were compared with those of meat in traditional polyethylene packaging. HENF significantly reduced drip and cooking loss as well as increased the moisture retention and quality of the meat. The antioxidant capacity of green tea extract was applied to reduce the lipid oxidation and increase the oxidative stability of beef. Incorporating HENF into the packaging at 3% increased the umami and richness taste traits of the meat, providing a novel approach for enhancing flavor profiles. Finally, HENF showed a high antimicrobial efficacy, reducing microbial counts and extending meat shelf life and safety.

Totaro and collaborators (Contribution 2) studied the use of inulin-based gels as fat replacers in beef burgers, optimizing formulations for improving the rheological properties of the meat. The gelling properties of high-degree-polymerization (HDP) inulin were enhanced compared with those of low-degree-polymerization (LDP) inulin. The authors emphasized the potential of using inulin to increase cooking yields and reduce shrinkage in meat products such as burgers. This approach addresses health concerns related to the fat content in meat products. A D-optimal mixture-process design was used to determine the optimal ingredient ratios, providing a novel approach to formulating fat replacers such as inulin. The optimal formulations were 51.52% inulin, 48.48% water, and 1.50% guar gum in the LDP gel. Furthermore, 39.12% inulin, 60.88% water, and 1.50% guar gum were optima for the HDP gel. Inulin gels maintained the functional quality of burgers while reducing the fat content, offering a healthier option for final consumers.

Deniz-González and collaborators (Contribution 3) studied the potential of using soursop (*Annona muricata* L.) leaf extracts as natural antioxidants in pork patties. They conducted preliminary research into the antioxidant activity and acute oral toxicity of hydroalcoholic extracts from soursop leaves. Higher concentrations of phenolic compounds were extracted using ethanol than using aqueous extractions, with the soursop showing synergistic extraction effects. Soursop leaf extract (20 g leaves/kg patty) may serve as a safe source of bioactive compounds, contributing to pork preservation through reducing oxidative and color damage, particularly in patties from Mexican rustic breeds. This study establishes a foundation for further research exploring the dose–response relationship, validating the applicability of soursop leaf extracts in food matrices, and studying rustic breeds in meat formulations.

Contribution 4 and Contribution 5 focused on the application of high-intensity ultrasound (HIU) on meat. HIU was explored as an alternative to chemical or thermal methods for processing meat due to the resulting changes in the physical and chemical properties of meat and meat products. These researchers considered the use of alternative meat species/breeds such as rabbits, guinea pigs, and rustic Mexican hairless pigs, as did Contributor 3, which reflects the necessity of exploring alternative species and breeds as meat sources.

Arteaga-Cabrera and collaborators (Contribution 4) found that HIU-assisted marination significantly enhanced the NaCl uptake and water-holding capacity in guinea pig meat compared with static marination, regardless of temperature. Short HIU applications (15 min) optimized the penetration of the marinade solution while preserving meat juiciness, whereas prolonged treatments led to muscle damage and increased weight loss. Additionally, controlled HIU treatment and low temperatures maximized marination efficiency and maintained sensory quality, such as the color attributes of the meat. The study highlights the effects of pH, as influenced by NaCl and acetic acid, on the moisture content, weight loss, and texture of meat. The authors suggested that future researchers should explore the effects of cooking on the sensory attributes and the impact of marination on the lipid oxidation and nutritional properties of guinea pig meat.

Contribution 5 found that HIU (20 min, 50 kHz, and 200 W) decreased the collagen content and shear force of rabbit meat, improving its physicochemical characteristics and the sensory perception of tenderness of rabbit meat (Flemish Giant, FG and Azteca Negro AN). Breed size did not influence meat tenderness, collagen content, or water holding capacity. However, the color profile and sensory perception were affected by the breed of the animal. HIU affected certain physicochemical properties; however, it did not compromise the overall quality of the meat.

Finally, the review by Khan and collaborators (Contribution 6) may seem challenging to the traditional meat industry; nevertheless, the article is highly valuable and relevant. Cultured meat represents a significant and alternative innovation, aimed at transforming food production, addressing both sustainability and health consumer concerns. Recent advances include innovations in cell selection, the optimization of culture media, and tissue engineering techniques. The role of biomaterials and bioreactors is critical in scaling up cultured meat production. Advances in stem cell biology allow for isolating cells from living animals, expanding cells in vitro, and directing their differentiation into muscle or fat cells. Outside of the process, other advances include considerable cost reductions for culture media and successful bioreactor expansions, although industrial scale-up and production remain limited. In addition, the main remaining challenges include replicating the nutritional and sensory properties of natural meat and developing serum-free media. Furthermore, interdisciplinary research is needed to integrate advancements from various fields, including cell biology, consumer perception, and regulations.

## 3. Conclusions

Research on meat is currently experiencing a substantial transformation, characterized by innovations aiming to improve not only the sensory quality and nutritional value of meat and meat products but also the sustainability of the production chain. Efforts are still underway to enhance traditional meat through innovations in muscle fiber modification using green technologies and applying protective natural ingredients. Alternatives are being studied, such as the development of cultured meat technologies to contribute to a more sustainable future. Furthermore, health-beneficial additives and environmental-friendly technologies are being developed in response to the increasing consumer demand for healthier meat, ethical production, and higher-quality food options. Ongoing research and development in these areas will redefine our approaches to meat production and consumption.
